# The implicit preference evaluation for the ceramic tiles with different visual features: Evidence from an event-related potential study

**DOI:** 10.3389/fpsyg.2023.1139687

**Published:** 2023-03-20

**Authors:** Jiayin Chen, Bingqin He, Huiqiu Zhu, Jianghua Wu

**Affiliations:** ^1^School of Design and Art, Jingdezhen Ceramic Institute, Jingdezhen, China; ^2^School of Ceramic Art, Jiangxi Arts and Ceramics Technology Institute, Jingdezhen, China; ^3^Department of Ophthalmology, Jingdezhen Third People's Hospital, Jingdezhen, China

**Keywords:** event-related potentials, implicit preference, ceramic tiles, visual features, N100, P200, N200

## Abstract

**Background:**

Ceramic tiles are popular because of their various forms, and they are often used to decorate the environment. However, few studies have applied objective methods to explore the implicit preference and visual attention of people toward ceramic tile features. Using event-related potential technology can provide neurophysiological evidence for the study and applications of tiles.

**Materials and methods:**

This study explored the influence of pattern, lightness, and color system factors of ceramic tiles on the preferences of people using a combination of subjective questionnaires and event-related potential (ERP) technology. Twelve different conditions of tiles (2 × 3 × 2) were used as stimuli. EEG data were collected from 20 participants while they watched the stimuli. Subjective preference scores and average ERPs were analyzed using analysis of variance and correlation analysis.

**Results:**

(1) Pattern, lightness, and color system factors significantly affected the subjective preference scores for tiles; the unpatterned tiles, light-toned tiles, and warm-colored tiles received higher preference scores. (2) The preferences of people for different features of tiles moderated ERP amplitudes. (3) The light-toned tiles with a high preference score caused a greater N100 amplitude than the medium-toned and dark-toned tiles; and the patterned tiles and warm-colored tiles with low preference scores induced greater P200 and N200 amplitudes.

**Discussion:**

In the early stage of visual processing, light-toned tiles attracted more attention, possibly because of the positive emotional effects related to the preference. The greater P200 and N200 elicited by the patterned and neutral-colored tiles in the middle stage of visual processing indicates that patterned and neutral-colored tiles attracted more attention. This may be due to negativity bias, where more attention is allocated to negative stimuli that people strongly dislike. From the perspective of cognitive processes, the results indicate that the lightness of ceramic tiles is the factor that people first detect, and the visual processing of pattern and color system factors of ceramic tiles belong to a higher level of visual processing. This study provides a new perspective and relevant information for assessing the visual characteristics of tiles for environmental designers and marketers involved in the ceramic tiles industry.

## 1. Introduction

As common decorative materials, tiles are widely used in both private and public environments. For example, ceramic tiles are the most popular materials for interior floor decoration (China Building Sanitary Ceramics Association, 2021). Tiles are not only representative of fashion but also provide a decorative environment through a combination of patterns, colors, and other design features (Albors-Garrigós et al., 2009). In daily life, people’s contact with ceramic tiles depends mainly on their vision (Artacho et al., 2020). Therefore, the design of tiles is primarily focused on the visual aspects. Some studies have correlated the visual features of design with human preferences (Mugge and Schoormans, 2012; Guo et al., 2019). For example, Agost and Vergara (2014) have found through questionnaires that the meaning and emotions (e.g., Well-being, calm, nervous.) contained in tiles with different features can influence the preferences of people. However, there are few studies on the preferences of tiles, especially on physiological responses (Chen and Cheng, 2022). Studying the preferences of people for tiles with different characteristics can help designers further improve the human experience.

Preference refers to how much people like a product (Zajonc and Markus, 1982; Roberts, 2007) and is crucial to the study of industrial design (Park et al., 2005). The preferences of people include an evaluation of the esthetic quality of the design. The object that people prefer often leads to a pleasant experience, whereas the object they do not prefer may lead to a negative experience (Palmer et al., 2013). Assessing the preferences of people for products can help improve product design and make products succeed in the competitive market (Macdonald et al., 2009; Orsborn and Cagan, 2009). Product form affects user preferences and plays an important role in purchasing decisions (Guo et al., 2016). The theory of Kansei engineering connects the formal characteristics of product design with the preferences of people and converts emotional preferences into words to guide product design (Nagamachi, 2002). Several important achievements have been made depending on Kansei Engineering. However, the commonly used methods, such as questionnaires and interviews, may not be able to obtain physiological evidence related to the preference (Ding et al., 2016). In recent years, many researchers have studied product preferences by evaluating the degree of preference for a specific product design appearance through physiological measurement methods (Wang et al., 2012; Ma et al., 2015). Research on preferences has gradually turned to more concretization. For example, Ma et al. (2015) found through an event-related potential (ERP) study that architectures with low preference scores attracted attention in the middle stage of visual processing. People’s preferred appearance of robots may attract attention in the early stages of visual processing (Guo et al., 2022). Therefore, specific experiments need to be conducted. In real life, most people do not make conscious preference judgments after seeing tiles; therefore, this study focused on people’s implicit preferences for different types of tiles. To avoid the influence of participant intention, this study chose an implicit preference assessment task (Wang et al., 2012).

Research on the preferences of tiles has received some attention. Serrano et al. (2013) found that participants’ satisfaction scores with the tiles significantly increased when they changed the tiles to their preferred type in an virtual reality research. Through a questionnaire survey, Agost and Vergara (2014) found that light-toned tile floors could bring people a happier sense of a bright and spacious environment compared to dark-toned tile floors. Therefore, this study evaluates the preference for tile floors with different features from the aspect of appearance. In a previous study, we found that different tile preferences could modulate neural activity. Specifically, the like-tiles induced more brain activity than dislike-tiles in the early visual stage. After that, the disliked-tiles induced more brain activity than the like-tiles in the middle and late visual stages (Chen and Cheng, 2022). Nevertheless, people may have different experiences with different tile types. For example, light-toned tiles bring people feelings of pleasure and relaxation (Agost and Vergara, 2014). Previous studies have shown that different levels of preference for tiles trigger differences in ERPs (Chen and Cheng, 2022). However, the preference and neural responses that people generate for tiles with different features are still unknown. Exploring the esthetic preferences and neural responses of ceramic tiles with different visual characteristics can help designers and researchers to understand people’s perceptions of ceramic tiles. Furthermore, the perception information about different tiles can help designers improve the design effect according to specific needs. Artacho et al. (2020) found through questionnaires that pattern, lightness, and color are the most important factors in terms of the human visual perception of tiles, thus identifying the three factors of tiles (pattern, lightness, and color) in this study. In contrast to our previous study (Chen and Cheng, 2022), the tiles in this study were applied to the indoor environment rather than presented as a single tile to make the participants feel more realistic. Furthermore, this study explores the influence of factors such as pattern, lightness, and color system.

In terms of research methods, most previous researchers have often used questionnaire as a quantitative research tool. Questionnaires are advantagesous because they afford a large sample size and can collect a large amount of information. However, participants’ responses are not always recorded instantly, and some responses may differ from their actual experiences, and the development of neurophysiology provides a more objective method of visual perception research for investigating people’s feelings (Ding et al., 2016; Zhang, 2020). In terms of neurophysiological measurement, La Parra-Hernandz et al. conducted a study using electromyogram (EMG) and galvanic skin reflex (GSR) techniques and found that different tiles could cause changes in arousal but not in valence (Laparra-Hernández et al., 2009). Nevertheless, some researchers have found that participants’ age, gender, body temperature, skin humidity, and respiratory rate tend to affect GSR results, leading to difficulties in comparing GSR results (Guo et al., 2019). In addition, the environment can significantly affect experimental results. Some researchers have used real environments for their experiments (Artacho et al., 2020). However, real environments require many resources, and interference factors in realistic experimental environments can affect research results. Therefore, this study was conducted in a laboratory setting.

ERP recorded by electroencephalography (EEG) has the advantages of high time precision and no trauma to participants, and it can be used for physiological measurements, which cannot be achieved by questionnaires or interviews (Daliri, 2013; Zeng et al., 2013; Jiang et al., 2018; Hou and Yang, 2021). Previous neurological studies on visual perception have indicated that human visual processing is usually related to mechanisms such as perception, attention, memory, reward, and emotional processing (Beudt and Jacobsen, 2015; Righi et al., 2017). The ERP technique performs well in measuring visual perception of stimuli (Bayer and Schacht, 2014; Lin et al., 2015). These results were obtained by studying the characteristics of different ERP components such as N100, P200, and N200.

Some early and middle ERP components can reveal attentional resource allocation (Song and Zhao, 2012). The N100 (a negative-going electrical potential, usually peaking around 100 ms after stimulation) is sensitive to low-level visual features and reveals the attentional allocation in perceptual processing (Luck et al., 2000; Vogel and Luck, 2000). Regarding P200 (a positive-going electrical potential that peaks around 100–200 ms after stimulation), many researchers have pointed out that it is correlated to visual perception (de Tommaso et al., 2008; Handy et al., 2010), and reveals the attentional allocation (Kosonogov et al., 2019). The N200 is related to automatic stimulus recognition, the formation of higher-order cognitive processes, and selective attention (Ernst et al., 2013; Li et al., 2013).

Emotion is a critical factor that affects people’s perceptions (Rolls, 2017). It strongly determines attractive and repulsive behaviors (Damasio and Carvalho, 2013; Righi et al., 2014). The cognitive process of esthetic preference involves the participation of emotions (Chatterjee, 2004; Brown et al., 2011). In affect-based assessments, preference is correlated with emotion (positive/pleasant or negative/unpleasant), which can produce different distributions of attention that are reflected in the amplitudes of the ERP components (Pessoa, 2008; Guo et al., 2022). Many researchers have demonstrated that a preference judgment for a design can influence attention formation and ERP amplitude (Handy et al., 2010; Beudt and Jacobsen, 2015; Cao et al., 2021).

For the tiles, in a previous ERP study, we found that the preference factor modulated the ERP amplitudes. For example, the N100 elicited by like-tiles is larger than that elicited by dislike-tiles (Chen and Cheng, 2022). In a previous study, ceramic tiles were divided into two categories (like-tiles or dislike-tiles). However, there are many types of tiles; therefore, the preferences of people toward different tile features (pattern, lightness, and color system factors) need to be explored. In addition, Guo et al. (2022) found that preferred robot appearances can induce greater N100 amplitudes than non-preferred robot appearances. Therefore, Hypothesis 1 was proposed. H1: The features of tiles (pattern, lightness, and color system factors) that people prefer elicit larger N100 amplitudes than those of tiles that people do not prefer.

Many studies have found that design preference can influence attentional allocation and P200 amplitudes (Righi et al., 2017; Chen and Cheng, 2022). For example, in a previous ERP study, we found that dislike-tiles elicited a larger P200 than like-tiles did in the posterior region of the brain (Chen and Cheng, 2022). Wang et al. (2012) found through an ERP study that P200 amplitudes can be effectively modulated by the preference of people for pendant design, and that ugly pendants induced larger P200 amplitudes. Ma et al. (2015) found that the architectures with low esthetic experience scores induced larger P200 amplitudes than those with high esthetic experience scores. In another ERP study on the preference for Chinese characters, Li et al. (2015) found that the preference factor significantly modulated the P200 amplitudes in the parietal and occipital regions. Thus, Hypothesis 2 was proposed. H2: The features of tiles that people do not prefer elicit a larger P200 amplitude than those elicited by prefer tile features in the parietal and occipital regions.

It has been demonstrated that N200 is associated with visual assessment (Folstein and Van Petten, 2008). In a study on preferences, N200 is considered an indicator of consumer preference (Vogel and Machizawa, 2004). In addition, many researchers have found that people’s preferred products triggered smaller N200 amplitudes in the frontal electrodes (de Tommaso et al., 2008; Telpaz et al., 2015; Goto et al., 2017). For example, Telpaz et al. (2015) found in product preference studies that a product with the highest preference score elicited the smallest N200. In contrast, the product with the lowest preference score elicited the highest N200. Goto et al. found that the N200 induced in the Fz electrode could predict people’s preferences relatively accurately (Goto et al., 2019). Therefore, Hypothesis 3 was proposed. H3: The features of tiles that people do not prefer elicit larger N200 amplitudes than those of tiles that people prefer in the frontal region.

This paper explores the preferences of people for different tile features, and how different features of the tile will affect cognitive processes (allocation of attention). To address these hypotheses, this study was organized into two research questions as follows:

1. Do different tile features lead to differences in subjective preferences?
2. Do different tile features cause differences in the ERP component amplitudes (N100, P200, and N200)?

Therefore, we conducted ERP experiments and used subjective questionnaires to explore the differences in the preferences of people with different tile features. At the time of the prevalence of COVID-19, studying the preferences of people for tile features may help designers effectively enhance their design so as to improve the experience of people working and living indoors.

## 2. Methods

### 2.1. Participants

We used G*Power 3.1 software to calculate the sample size. When α was 0.05 and the power (1-β) was 0.95, a minimal total sample size of 18 was required to detect a medium effect size of 0.25. Based on similar ERP researches (Beudt and Jacobsen, 2015; Guo et al., 2019; Chen and Cheng, 2022), we selected 20 undergraduates (11 females and 9 males, 18–31 years old, average age 22.5) as participants in the present study. All the participants were right-handed and had normal visual or corrected visual acuity. To avoid the health problem of participants affecting the results of the study, we asked clinicians to inspect participants for a history of neurological and psychiatric disorders, autoimmune disease, major depression, and mild cognitive impairment. None of the participants in this study had a history of brain injury, systemic disease, rheumatic disease, or autoimmune disease. In addition, all the participants were asked to rest well and not take stimulants or psychotropic drugs before the experiment. After the experiment, each participant was paid 80 CNY.

### 2.2. Stimuli

**Figure 1:**
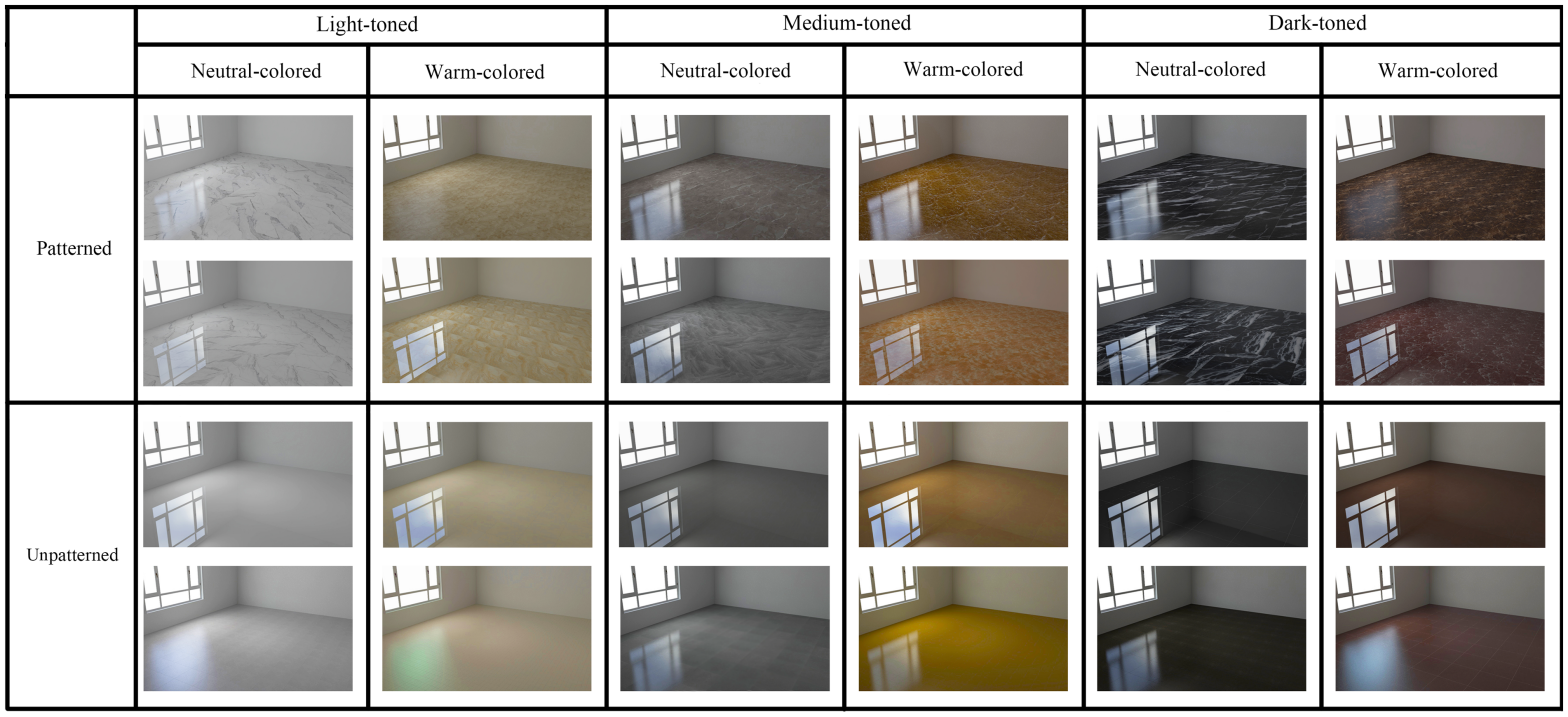
Stimuli samples of 12 different types of tiles.

Gao et al. (2019) found in the environmental research that no significant differences between the experience of field investigation and that of images, which confirms that images can be used in experimental research as stimuli on environmental perception. A previous study also demonstrated that experimental studies could be conducted using neuroscience techniques with 2D picture stimulation of tiles (Chen and Cheng, 2022). Therefore, pictures of the tiles were used as the stimuli. In an event-related potential study, Wan et al. (2021) classified the lightness of stimuli into light, medium, and dark levels, and found that the lightness factor can modulate brain activity. However, the effect of tile brightness on brain activity is still unknown. In terms of color, Zhang et al. (2020) pointed out that tiles with neutral and warm color systems are the most common in the tiles market. In terms of pattern, Zhang et al. (2020) pointed out that tiles on the market are usually divided into patterned and unpatterned types. Therefore, this present study classified the lightness of tiles into three levels: light-toned, medium-toned, and dark-toned (Wan et al., 2021). The three levels of lightness were differentiated according to the brightness level of the ceramic tile color. For example, white and beige tiles are considered light-toned, gray and yellow-brown tiles are medium-toned, and black and dark brown tiles are dark-toned. The color factor is divided into two levels: neutral-colored and warm-colored (Zhang et al., 2020). The pattern factor is divided into two levels: patterned and unpatterned (Zhang et al., 2020). The pattern of the ceramic tile is composed of a decorated texture on its surface. When the surface of the ceramic tile is decorated with points, lines, and planes, it is regarded as a patterned ceramic tile. When the surface of the ceramic tile is not decorated with points, lines, and planes, it is regarded as a unpatterned tile. It should be emphasized that the pattern of tiles described here is flat and two-dimensional; therefore, it does not need to be perceived through touch. In summary, 12 different tile conditions (two levels of pattern factor × three levels of lightness factor × two levels of color system factor) were applied in this study, with two tiles of each condition for a total of 24 tiles (Figure 1). The purpose of using two tiles per condition was to avoid people’s preference for a single tile, thus influencing the results of each type. Stimulus pictures were rendered using 3Dmax software. A neutral room was used as the environment to analyze the preferences for different tiles. No furniture or decoration was added to the room to avoid distractions. The resolution of the images was uniformly set to 1,280 × 768 pixels and was displayed on a 15.6-inch LCD screen (1,280 × 768, 60 Hz).

### 2.3. Procedure

**Figure 2:**
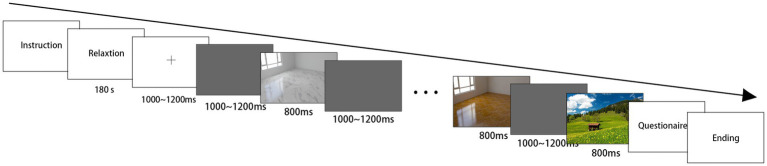
Task paradigm with the timing of presentation.

The main purpose of this experiment was to explore the differences in implicit preferences and automatic attentional allocation caused by different features of ceramic tiles. Therefore, we used a modified oddball paradigm (Ma et al., 2018; Guo et al., 2019; Chen and Cheng, 2022) in which participants were asked to look at different pictures of tile floors without making a decision. Pictures containing tile floors were presented as frequent non-target stimuli, and landscape pictures were presented as rare target stimuli. All the participants sat 60 cm from the front of the computer screen to view the stimulus pictures with a viewing angle of approximately 32.9° × 18.5° (width × height). The ERP task was programmed and presented using E-prime 2.0, and each picture of the 12 types of tiles was repeated 40 times. Stimuli were presented randomly to eliminate the order effect, as shown in Figure 2. First, a 3-min countdown was used to facilitate participants’ relaxation, and then a plus sign appeared to help participants focus on the center of the picture, followed by the alternating presentation of the stimuli. Each image was displayed for 800 ms, followed by a gray screen for 1,200 ms to return the participants’ visual perception to baseline. After the ERP experiment, each participant was asked to complete a questionnaire that reflected their subjective preferences for each stimulus. Each experiment lasted about 40 min, with three breaks in between.

### 2.4. Subjective questionnaire

Subjective questionnaires are typically used to measure the preferences for different tiles. Wang et al. (2012) asked participants to evaluate their degree of preference for pendant design to collect product preferences using a 7-point Likert scale. de Tommaso et al. (2008) asked participants to rate the appearance of experimental stimuli to assess the level of stimulus preference using a 10-point Likert scale. Therefore, this study used a questionnaire to measure the preferences of people for different tiles. The question was: “From the aspect of appearance preference, do you like or dislike the tiles?” The indicator was divided into two: like and dislike. Participants were asked to rate the esthetic preference of different tiles separately on a 9-point Likert scale, ranging from 1 to 9, where 1 indicates intensely disliked and 9 indicates intensely liked.

### 2.5. Electrophysiological recording and analysis

**Figure 3:**
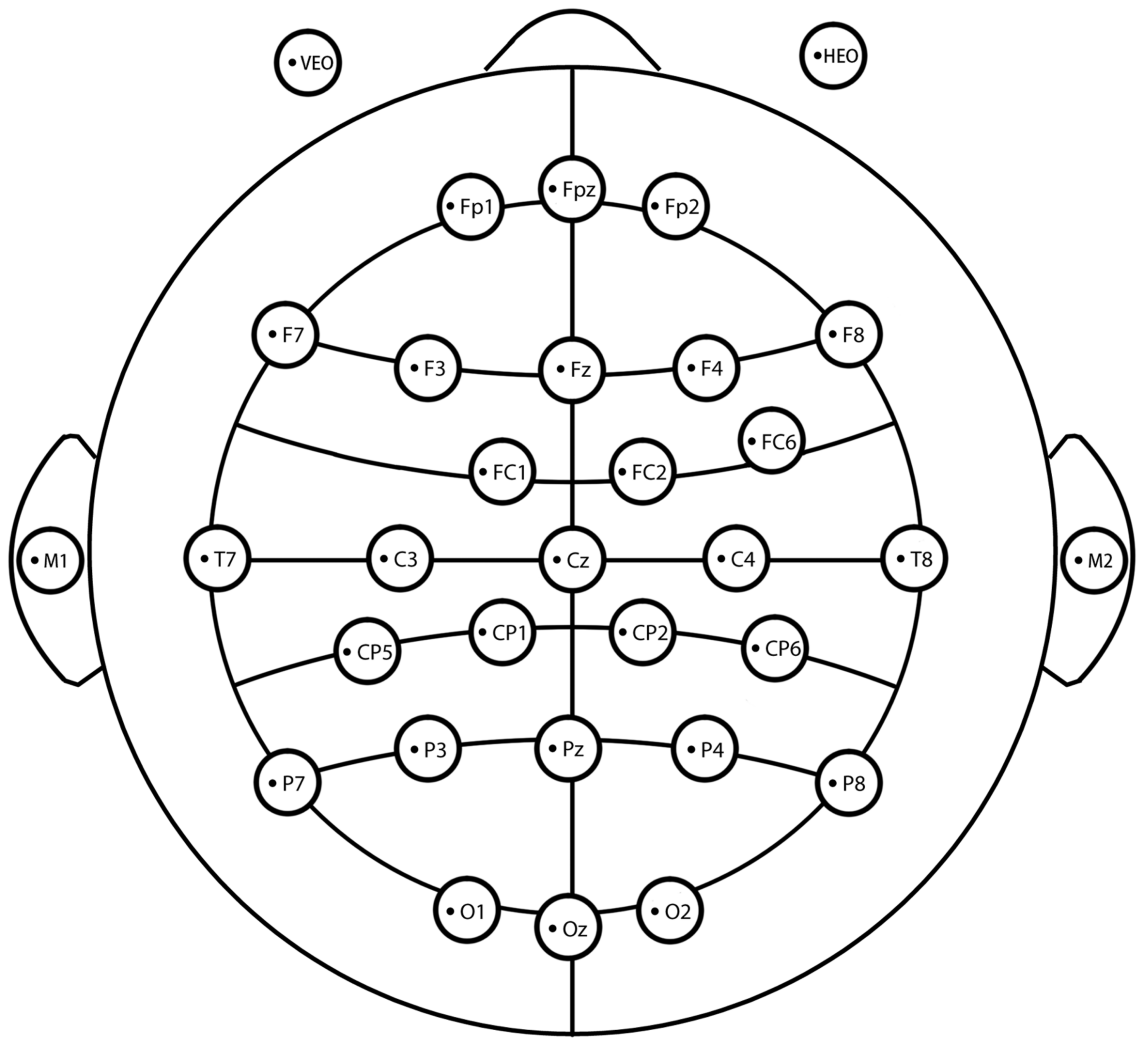
A diagram of the electrodes used in the experiment.

A SMARTING PRO EEG system (32 electrodes) was used to continuously record EEG signals in this study. According to an extended version of the international 10–20 electrode placement system (Figure 3), the electrodes were located at 32 standard positions [Fp1/2, Fpz, F3/4, F7/8, Fz, Fc1/2, Fc6, C3/4, T7/8, Cz, Cp1/2, Cp5/6, P3/4, P7/8, Pz, O1/2, Oz, vertical electrooculogram (VEOG) and horizontal electrooculogram (HEOG), M1/2]. The midpoint of Fz and Fpz was used as the ground electrode, and the reference electrodes (M1 and M2) were placed at the bilateral ear lobes. The VEOG was placed at the infraorbital region of the right eye, and the HEOG was placed at the outer canthi of the left eye. The impedance of each electrode was less than 5kΩ. After recording, an offline pretreatment was conducted using the EEGLAB toolbox. The procedure was divided into the following steps: 1. Remove useless electrodes (eye electrodes); 2. Filter at 0.1 ~ 30 Hz; 3. Segment processing (−200 ms ~ 800 ms); 4. Re-reference with the average of the earlobe electrodes; 5. Independent component analysis (ICA); 6. Manually identification and deletion of artifacts; and 7. Stack and average ERPs. Stimulation of each condition was repeated at least 60 times in the data retained after pretreatment.

Based on previous studies (Chatterjee and Vartanian, 2014), the N100 amplitudes at the electrodes of the frontal (Fz, F3, and F4), central (Cz, C3, and C4), parietal (Pz, P3, and P4), and occipital (Oz, O1, and O2) regions were included in the statistical analysis. Previous studies have shown that the parietal and occipital regions are usually chosen for the analysis of P200 (Guo et al., 2022; Liu et al., 2022). In addition, the waveform plots and topographic maps elicited by the pattern, lightness, and color system factors in this experiment showed that the P200 amplitudes elicited in the frontal and central regions were not obvious. Therefore, the P200 amplitudes in the parietal and occipital regions (Pz, P3, P4, Oz, O1, and O2) were chosen for analysis. For the N200, many authoritative studies have shown that the N200 component is mainly evident at the frontal and central electrodes (Folstein and Van Petten, 2008; Telpaz et al., 2015). Moreover, combined with the waveform plots of our experiment, the N200 amplitudes on parietal and occipital electrodes were not obvious. Therefore, we chose the frontal and central regions (Fz, F3, F4, Cz, C3, and C4) for analysis. The time window was taken as the period of time around the peak of each grand average ERP (Chen and Cheng, 2022). For the N100, the amplitudes in the frontal, central, parietal, and occipital regions were used for analysis in a time window of 80–130 ms. For the P200, the amplitudes in the parietal and occipital regions were used for analysis in a time window of 200–260 ms. For the N200, the amplitudes in the frontal and central regions were used for analysis in a time window of 260–330 ms. The average amplitude of each time window was used as the dependent variable for repeated-measures ANOVA. Each ANOVA included four independent variables: lightness (light-toned, medium-toned, or dark-toned), pattern (patterned or unpatterned), color system (neutral-or warm-colored system), and electrode (frontal, central, parietal, or occipital regions). Pearson correlation analysis was used to compare the subjective preference scores and ERP amplitudes.

All statistical analyses were conducted using SPSS Statistics 25 for statistical significance testing and were considered statistically significant at *p* < 0.05. The analyzed data in SPSS were corrected using Greenhouse–Geisser.

## 3. Results

The goal of this study was to investigate the preferences of people for different tile features. The results of subjective evaluation (preference) and ERP amplitudes (N100, P200, and N200) are reported below.

### 3.1. Subjective evaluation

Repeated-measures ANOVA showed that pattern [*F* (1,19) = 4.455, *p* = 0.048, partial η^2^ = 0.19], lightness [*F* (2,38) = 32.188, *p* < 0.001, partial η^2^ = 0.629], and color system [F (1,19) = 16.125, *p* = 0.001, partial η^2^ = 0.459] had significant effects on preference ratings. Participants preferred unpatterned tiles (mean = 5.042, SD = 0.209) over patterned tiles (mean = 4.458., SD = 0.233). The preference rating score of light-toned tiles (mean = 6.075, SD = 0.298) was higher than that of medium-toned tiles (mean = 4.638, SD = 0.253) and dark-toned tiles (mean = 3.537, SD = 0.195). The preference score of warm-colored tiles (mean = 5.158, SD = 0.193) was higher than that of neutral-colored tiles (mean = 4.342, SD = 0.209). Table 1 presents the details of the ANOVA results.

**Table 1:** ANOVA of subjective preference scores for the different features of tiles.

Factors	*F*	*P*	Partial η^2^	Levels	Preference score	
					Mean	SD
Pattern	4.455	0.048	0.19	Patterned	4.458	0.233
				Unpatterned	5.042	0.209
Lightness	32.188	<0.001	0.629	Light-toned	6.075	0.298
				Medium-toned	4.638	0.235
				Dark-toned	3.537	0.195
Color system	16.125	0.001	0.459	Neutral-colored	4.342	0.209
				Warm-colored	5.158	0.193

### 3.2. Event-related potentials

**Figure 4:**
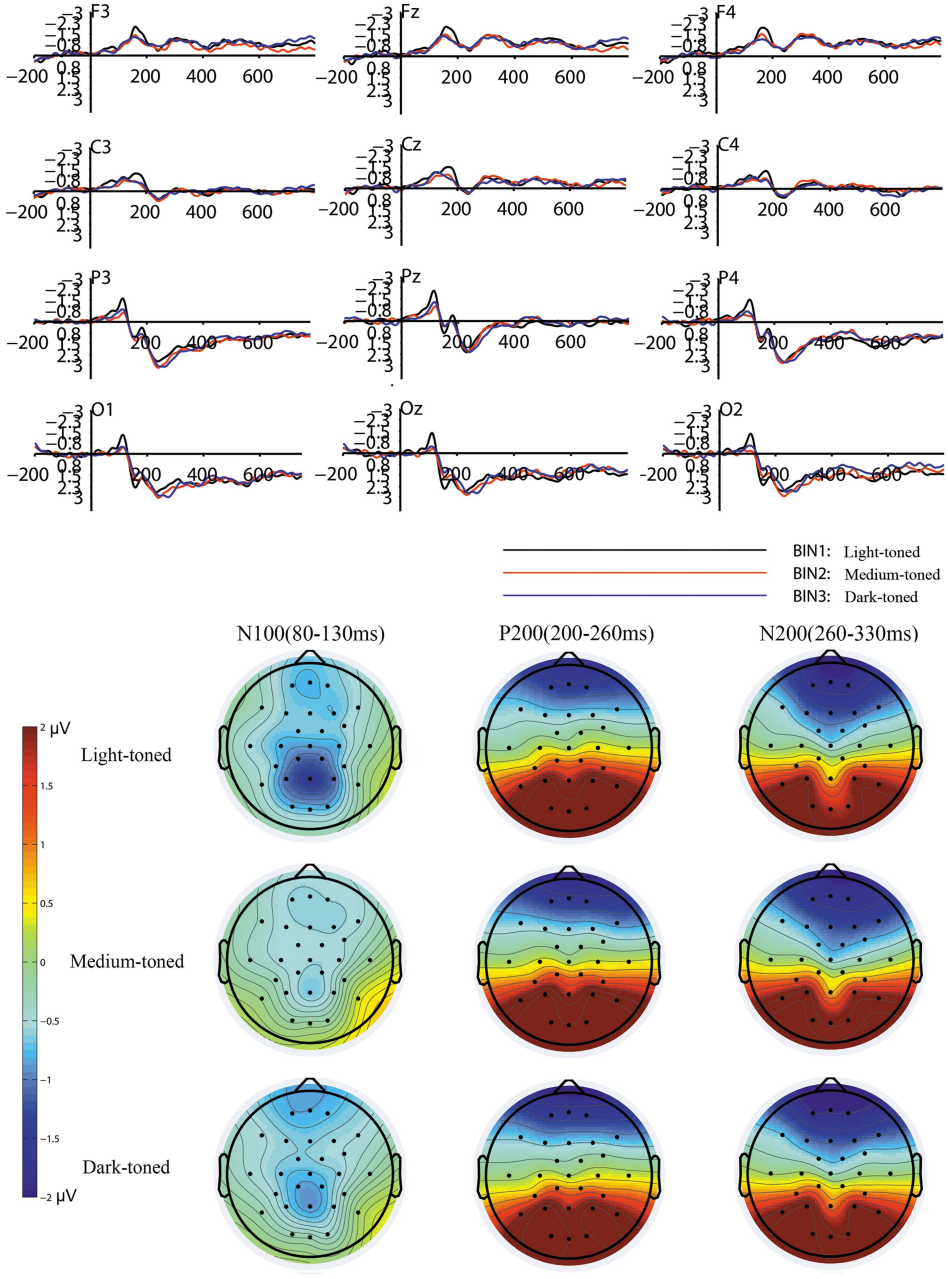
The grand averaged waveforms and topography map for the three conditions of the lightness factor.

Repeated-measures ANOVA showed that the lightness factor of tiles had a significant effect on the N100 (80–130 ms) amplitudes in the frontal, central, parietal and occipital regions [*F* (2, 38) = 4.218, *p* = 0.024, partial η^2^ = 0.182]. There was no significant effect of pattern [*F* (1, 19) = 0.578, *p* = 0.456, partial η2 = 0.03] or color system [F (1, 19) = 1.157, *p* = 0.296, partial η^2^ = 0.057] factors. The interaction between lightness, pattern, color system and electrode factors was insignificant [*F* (2, 38) = 3.206, *p* = 0.053, partial η2 = 0.144]. The electrode factor had no significant effect on N100 [*F* (11, 209) = 1.938, *p* = 0.134, partial η^2^ = 0.093]. The mean N100 amplitude elicited by the light-toned tiles (mean = −0.9, SD = 0.186) was lower than that elicited by the medium-toned tiles (mean = −0.579, SD = 0.156) and dark-toned tiles (mean = −0.7, SD = 0.15). The results of the Pearson correlation analysis showed a significant negative correlation between the mean value of the subjective preference scores and the mean N100 amplitudes (r = −0.607, *p* = 0.005). Table 2 presents more details of the ANOVA results. The grand average waveforms and topography map caused by the different lightness levels of the tiles are shown in Figure 4.

**Table 2:** ANOVA of ERP amplitudes for the different features of tiles.

ERP	Region	Factors	*F*	*P*	Partial η^2^	Levels	Preference score	
							Mean	SD
N100	Frontal, central, parietal, and occipital	Ligntness	4.218	0.024	0.182	Light-toned	−0.9	0.186
						Medium-toned	−0.579	0.156
						Dark-toned	−0.7	0.15
P200	Parietal and occipital	Pattern	17.198	0.001	0.475	Patterned	2.96	0.61
						Unpatterned	2.141	0.523
		Color system	5.891	0.025	0.237	Neutral-colored	2.715	0.58
						Warm-colored	2.386	0.547
N200	Frontal	Pattern	10.059	0.005	0.346	Patterned	−1.521	0.321
						Unpatterned	−0.886	0.244
		Color system	11.324	0.003	0.373	Neutral-colored	−1.462	0.272
						Warm-colored	−0.945	0.283
	Central	Pattern	6.361	0.021	0.251	Patterned	−0.469	0.24
						Unpatterned	−0.089	0.201

**Figure 5:**
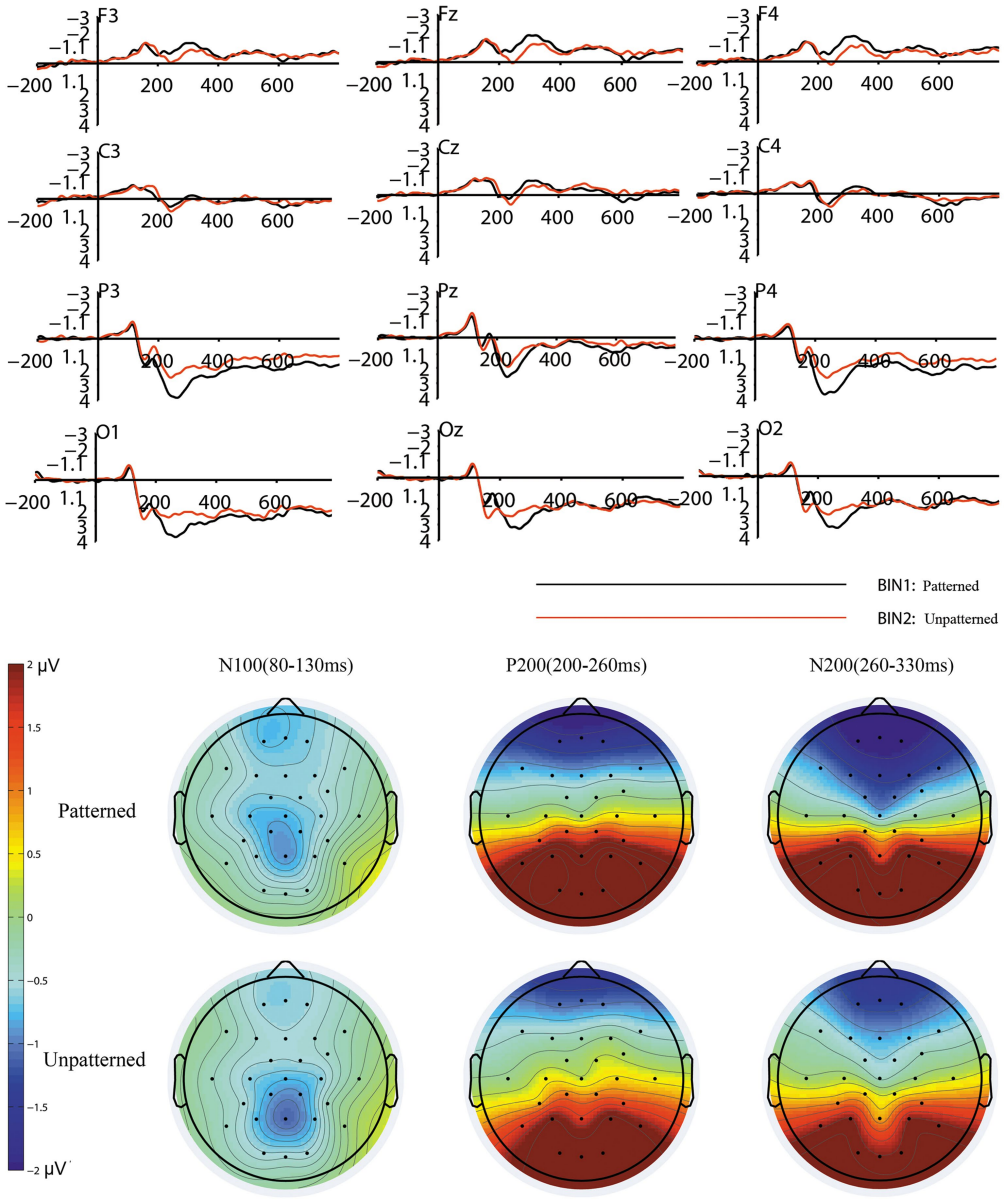
The grand averaged waveforms and topography map for the two conditions of the pattern factor.

**Figure 6:**
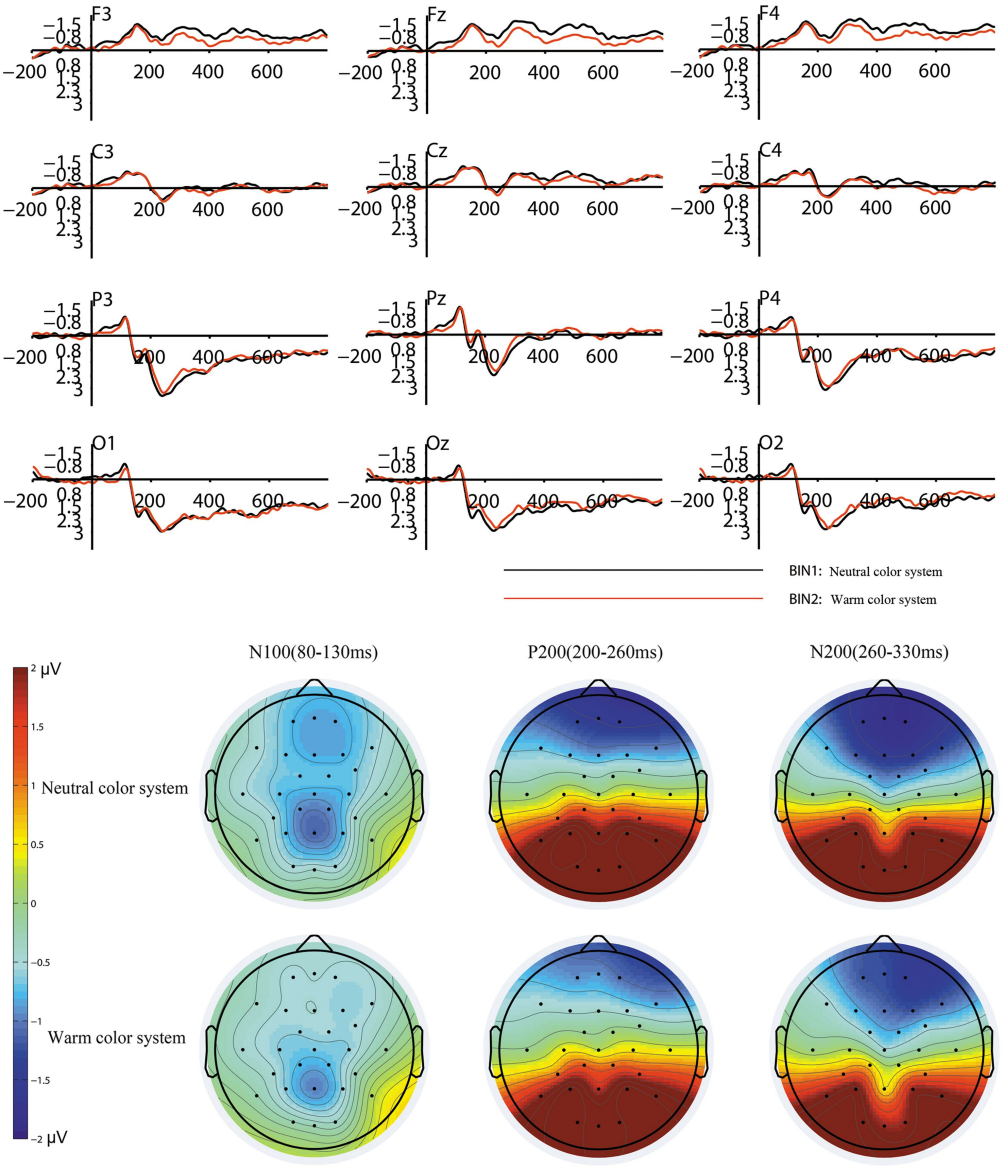
The grand averaged waveforms and topography map for the two conditions of the color system factor.

For P200 (200–260 ms), repeated-measures ANOVA showed that the pattern factor [*F* (1, 19) = 17.198, *p* = 0.001, partial η^2^ = 0.475] and color system [*F* (1, 19) = 5.891, *p* = 0.025, partial η^2^ = 0.237] factors had significant effects on P200 in the parietal and occipital regions. There was no significant effect of the lightness factor [F (2, 38) = 0.876, *p* = 0.423, partial η^2^ = 0.044] on the P200 amplitudes. The interaction between the pattern, lightness, and color system factors was not significant [F (2, 38) = 0.038, *p* = 0.961, partial η^2^ = 0.002]. The electrode factor had no significant effect on P200 [*F* (5, 95) = 1.325, *p* = 0.275, partial η^2^ = 0.065]. The mean P200 amplitude that elicited by patterned tiles (mean = 2.96, SD = 0.61) was higher than that elicited by unpatterned tiles (mean = 2.141, SD = 0.523). The mean P200 amplitude that elicited by neutral-colored tiles (mean = 2.715, SD = 0.58) was higher than that elicited by warm-colored tiles (mean = 2.386, SD = 0.547). There was a significant negative correlation between the mean value of the subjective preference scores and the mean P200 amplitudes (*r* = −0.629, *p* = 0.003). The grand average waveforms and topography map caused by different pattern levels of tiles are shown in Figure 5. The grand average waveforms and topography map caused by different color system levels of tiles are shown in Figure 6.

For N200 (260–330 ms), repeated-measures ANOVA showed significant effects of pattern [*F* (1, 19) = 9.134, *p* = 0.007, partial η^2^ = 0.325] and color system [*F* (1, 19) = 6.195, *p* = 0.022, partial η^2^ = 0.246] factors on the frontal and central regions. There was no significant effect of the lightness factor [*F* (2, 38) = 0.06, *p* = 0.936, partial η2 = 0.003]. There was no significant interaction between the pattern, lightness, and color system factors [*F* (1, 19) = 0.81, *p* = 0.537, partial η^2^ = 0.041]. The electrode factor had a significant effect on N200 [*F* (5, 95) = 22.974, *p* < 0.001, partial η^2^ = 0.547]. The pattern factor induced significant effects on the N200 amplitudes in the frontal [F (1, 19) = 10.059, *p* = 0.005, partial η^2^ = 0.346] and central [F (1, 19) = 6.361, *p* = 0.021, partial η^2^ = 0.251] regions. The color system factor induced significant effects on the N200 amplitudes in the frontal region [F (1, 19) = 11.324, *p* = 0.003, partial η^2^ = 0.373], but had an insignificant effect on the N200 amplitudes of the central region [*F* (1, 19) = 0.936, *p* = 0.345, partial η^2^ = 0.047]. The mean N200 amplitude in the frontal region that elicited by patterned tiles (mean = −1.521, SD = 0.321) was lower than that elicited by unpatterned tiles (mean = −0.886, SD = 0.244). The mean N200 amplitude in the central region that elicited by patterned tiles (mean = −0.469, SD = 0.24) was lower than that elicited by unpatterned tiles (mean = −0.089, SD = 0.201). The mean N200 amplitude in the frontal region that induced by neutral-colored tiles (mean = −1.462, SD = 0.272) was lower than that induced by the warm-colored tiles (mean = −0.945, SD = 0.283). There was a significant positive correlation between the mean value of subjective preference scores and mean N200 amplitudes in the frontal and central regions (*r* = 0.688, *p* = 0.001).

## 4. Discussion

This study applied a combination of ERP and self-reporting methods to study the preferences of people for different features of tiles, and the subjective preferences and neural responses of people for different ceramic tiles were collected.

The results showed that light-toned tiles that people preferred elicited larger N100 amplitudes. N100 is sensitive to brightness and reflects automatic attentional resource allocation (Anllo-Vento and Hillyard, 1996; Vogel and Luck, 2000). Based on these studies, the light-toned tiles induced the greatest N100 amplitude in the parietal and occipital regions in our study, possibly indicating that the light-toned tiles attracted more attentional resources than the medium-toned and dark-toned tiles in the early stage of visual processing. Many studies have revealed that the preference judgment for the appearance of a design could influence attention formation and ERP amplitudes (Wang et al., 2012; Chen and Cheng, 2022; Guo et al., 2022; Liu et al., 2022). The preference formation theory points out that the appearance of objects that people prefer can attract users and have a positive influence (Zajonc and Markus, 1982). In addition, some researchers have found that not only does bright stimulus cause a larger difference in N100 amplitude compared to dim stimuli, but the N100 latency induced by bright stimuli is significantly shorter than that induced by dim stimuli (Carillo-de-la-Peña et al., 1999; Johannes et al., 2003). However, there was no significant difference in the N100 latency between light-toned, medium-toned and dark-toned tiles in this study. Therefore, the differences in the N100 components in our experiment may not be induced by changes in stimulus brightness intensity, but rather by the preference-related attention allocation. Thus, the positive influence of preference prompts people to pay more attention to the preferred stimulus during early visual processing stages and is reflected in the larger N100 amplitude, which is consistent with previous studies (Handy et al., 2010; Guo et al., 2022). The result demonstrating that the light-toned tiles with the highest preference scores induced the largest N100 amplitudes and the negative correlation between preference scores and N100 amplitude supported the view that the tile features that people preferred can induce a greater N100 than those that are not preferred. These results support H1. Another interpretation is that N100 may reflect emotions induced by stimuli, as Keil et al. (2001) pointed out in a previous study. Agost and Vergara (2014) pointed out that the emotion contained in the tiles can influence the preferences for tiles. Although the difference in N100 amplitude caused by dark-and medium-tone tiles was insignificant, both light-and dark-toned tiles induced larger N100 than medium-toned tiles, confirming the view that both pleasant and unpleasant stimuli trigger larger N100 than neutral stimuli (Keil et al., 2001). The N100 induced by light-toned tiles is the largest, that may be because the pleasure induced by light-toned tiles is strong, and the unpleasant feelings induced by dark-toned tiles is not strong. This result is consistent with the study by Agost and Vergara that people preferred light-toned tiles because light-toned tiles make the environment feel more spacious, bright, and comfortable (Agost and Vergara, 2014). In terms of temporal order, the pattern and color system did not induce a difference in the N100 amplitudes, indicating that the first thing that the participants could distinguish might be the lightness factor of the tiles. The lag of the differences in the amplitude generated by the pattern and color system factors may indicate that these two factors require more time to differentiate and are related to higher-order cognitive processes.

The ANOVA results revealed that the patterned and neutral-colored tiles with low preference scores elicited larger P200 values in the parietal and occipital regions. P200 is associated with higher-order perceptual processing and attention allocation (Herbert et al., 2006; Yuan et al., 2007; de Tommaso et al., 2008). Many researchers have suggested that the P200 amplitude in the posterior brain region indicates a larger allocation of automatic attentional resources to negative stimuli (Dennis and Chen, 2007; Lin et al., 2018). This phenomenon may be caused by the negativity bias that stimuli are automatically processed to be more emotionally arousing when they make people feel unpleasant and then attract people’s automatic attention (Righi et al., 2017). In neurological studies of product design, many studies have found that an increase in P200 amplitude correlates with the negativity bias that the non-preferred designs evoke significantly greater P200 than those elicited by preferred designs (Wang et al., 2012; Ma et al., 2015; Chen and Cheng, 2022). This is also confirmed by the results of our correlation analysis; when people’s preference value is lower, the P200 amplitude is larger. According to the interpretation of the negativity bias and previous studies (Wang et al., 2012; Ma et al., 2015; Chen and Cheng, 2022), patterned and neutral-colored tiles induced greater P200, which may be due to the automatic negativity bias in which the preferential processing of negative stimuli affects attention allocation (Cacioppo and Berntson, 1994; Simpson et al., 2000). Therefore, we suggest that patterned and neutral-colored tiles that people do not prefer attract more attentional resources. These results support H2. The dark-toned tiles did not cause a larger P200 amplitude. This may be because the unpleasant feelings induced by the dark-toned tiles are not strong, which is also reflected in the N100 amplitude. Patterned tiles are preferred less than unpatterned tiles, which may be because the unpatterned tiles look cleaner and neater (Agost and Vergara, 2014). Jonauskaite et al. found that people prefer neutral colors less, which may be because neutral colors appear to be less chromatic and tend to be more negative (Jonauskaite et al., 2019). Therefore, we suggested that participants preferred warm tones, possibly because warm tones are usually associated with positivity and can create a warm and cozy perception of the environment. In addition, the difference in the amplitudes of the P200 induced by the pattern and color system factors confirms the previous view that people’s discrimination against these two factors takes more time than lightness factors and is a higher-order cognitive process.

Following P200, patterned and neutral-colored tiles with low preference scores induced a greater N200 in the frontal region. Many studies have confirmed that preference can moderate N200 amplitudes and that low-preferred stimuli can elicit higher N200 amplitudes (de Tommaso et al., 2008; Telpaz et al., 2015; Goto et al., 2017; Lin et al., 2018). In this study, the positive correlation between preference scores and N200 amplitudes indicated that the features of tiles with higher preference scores elicited a smaller negative N200 deflection. The results are consistent with the conclusions of the preference prediction model proposed by Telpaz et al. (2015) and Goto et al. (2017), which showed that the N200 can predict the preferences of people. This phenomenon may be related to a negativity bias (Cacioppo and Berntson, 1994; Simpson et al., 2000; Dennis and Chen, 2007). Olofsson et al. (2008) pointed out that unpleasantness induced by stimuli could elicit greater N200 than pleasant stimuli in the anterior cingulate cortex. Thus, we suggest that the non-preferred tiles (patterned and neutral tiles) induced greater N200 amplitudes, reflecting a negative stimulus-driven attentional response, which is consistent with prior studies (Olofsson et al., 2008; Ma et al., 2010; Walker et al., 2011; Ding et al., 2017). These results support H3.

## 5. Conclusion

The goal of this study is to investigate the preferences of people for different tiles. Light-toned tiles with high preference induced greater N100 amplitudes than medium-toned and dark-toned tiles; the patterned and neutral-colored tiles with low preference induced greater P200 and N200 amplitudes. From a sequential point of view, the late appearance of neural response differences induced by the pattern and color system factors compared to the lightness factors may indicate that the lightness of tiles is the first factor to be distinguished. The visual processing of the pattern and color system factors of tiles belongs to a higher visual cognitive process that requires more time to distinguish. From the perspective of the allocation of attentional resources, the results indicate that in the early stage of visual processing (N100), the features of the tiles that people preferred (light-toned) attracted more attention, whereas in the middle stage of visual processing (P200 and N200), disliked features of the tiles (patterned and neutral-colored) attracted more attention. The correlation between preference scores and ERPs further validates the feasibility of using ERP techniques to measure the preferences for tiles. In terms of theoretical implications, this study reports the relationship between the preferences of people for different tile features and ERPs, which offers a new perspective for the study of neuro-esthetics and neurodesign. From a practical perspective, this study found that people prefer unpatterned, light-toned, and warm-colored tiles. This provides references for interior designers, environmental designers, and other relevant people. This study can provide researchers with a better understanding of the preferences for different tiles and help designers choose appropriate tiles for varied environments.

## 6. Limitations and future research

The study had three main limitations. First, although the number of participants in our experiment reached the minimum standard required for this study, including more participants would be preferable. Therefore, we will recruit more participants in future studies. Second, most of the participants were young students, and the preferences of different age groups may vary. Therefore, future studies should recruit multiple age groups. In addition, LPP correlates with the perceptual evaluation of a stimulus and may reflect the top-down allocation of motivational attention to emotional stimuli (Hou and Lu, 2018). However, the preference judgment in our study was implicit, and we aimed to explore the automatic attentional allocation induced by different tile features. During the ERP experiment, participants did not consciously evaluate the preferences for each stimulus. The lack of conscious judgment may explain why our experimental stimuli did not elicit significant differences in the LPP amplitudes. Behavioral data also contains a lot of useful information; hence, we will collect behavioral data about the preference judgment of people in future studies.

## Data availability statement

The raw data supporting the conclusions of this article will be made available by the authors, without undue reservation.

## Ethics statement

The studies involving human participants were reviewed and approved by the ethics committee of Jingdezhen Third People’s Hospital, China (LL2022003), and followed the Declaration of Helsinki. The patients/participants provided their written informed consent to participate in this study.

## Author contributions

JC was involved in study design, execution of experimental procedures, data analysis, and manuscript drafting and revision. BH was involved in manuscript revision. HZ was involved in study design. JW participated in the execution of part of the experimental procedures. All authors contributed to the article and approved the submitted version.

## Funding

This work is supported by the National Social Science Foundation (No. BJA190105), Jiangxi University Humanities and Social Sciences Research Project (No. YS20243), Science and technology research project of the Education Department of Jiangxi Province (No. 191303), and the Jiangxi Province Graduate Innovation Special Fund project (No. YC2021-B155).

## Conflict of interest

The authors declare that the research was conducted in the absence of any commercial or financial relationships that could be construed as potential conflicts of interest.

## Publisher’s note

All claims expressed in this article are solely those of the authors and do not necessarily represent those of their affiliated organizations, or those of the publisher, the editors and the reviewers. Any product that may be evaluated in this article, or claim that may be made by its manufacturer, is not guaranteed or endorsed by the publisher.
